# Supplementary material for: Systemic signaling contributes to the unfolded protein response of the plant endoplasmic reticulum
Source: Nat Commun. 2018 Sep 25;9:3918. doi: 10.1038/s41467-018-06289-9 (PMC6156401; doi:10.1038/s41467-018-06289-9)
Supplement: Supplementary file 1 — Supplementary Information [file 41467_2018_6289_MOESM1_ESM.pdf]

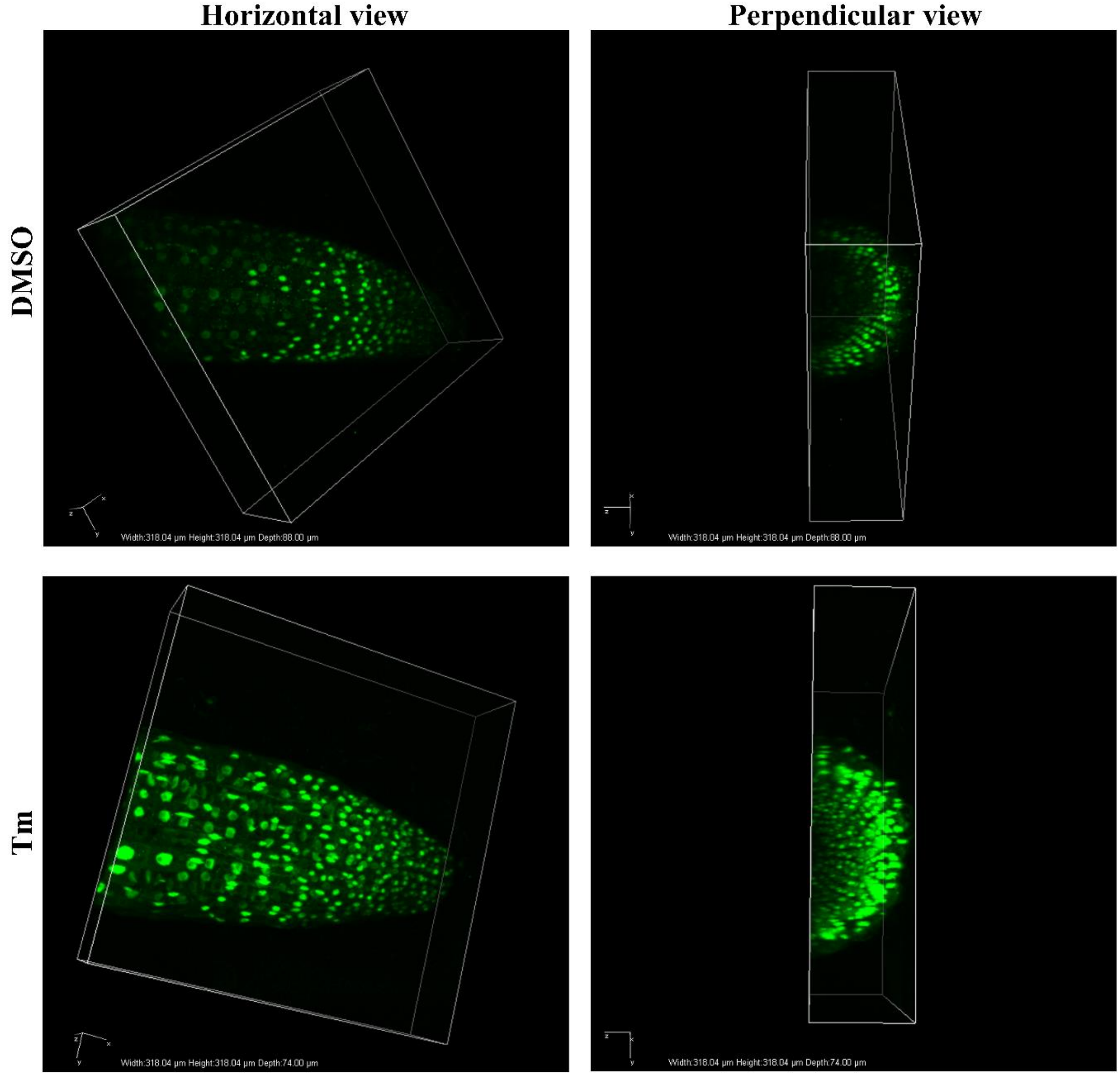

Supplementary Figure 1. Subcellular localization of GFP-bZIP60 in the root under physiological conditions and induced ER stress conditions.

Three-dimensional rendering of confocal optical sections of the root tips of 7-day-old *Arabidopsis bzip60*; *pbZIP60-GFP-bZIP60*[1] seedlings treated with DMSO or 0.5µM Tm for 24 hrs.

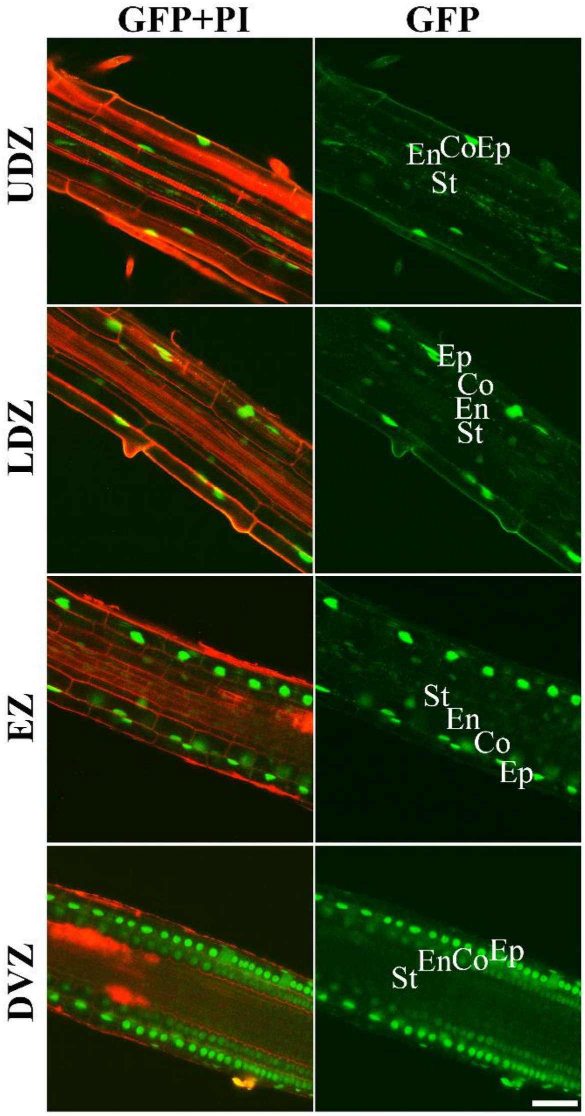

Supplementary Figure 2. Stele-expressed sbZIP60 distributes throughout the root

Confocal laser scanning microscopy analyses of 5-day-old *bzip28/60*; *pSHR-sbZIP60-GFP* throughout the primary root, including division zone (DVZ), elongation zone (EZ), lower differentiation zone (LDZ) and upper differentiation zone (UDZ), reveals a subcellular localization of sbZIP60-GFP in the nuclei of stele (St), endodermis (En), cortex (Co) and epidermis (Ep). Scale bar: 50 μm.

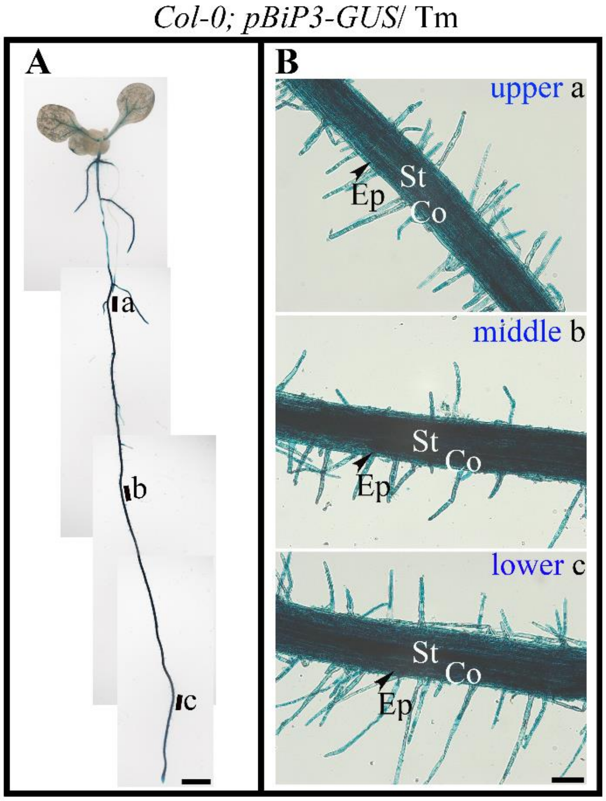

Supplementary Figure 3. ER stress-induced *BiP3* expression occurs throughout the root

(A) Expression of *pBIP3:GUS* in 11-day-old Col-0 seedlings treated with 0.5μM Tm for 24 hrs. X-Gluc was used for histochemical staining to monitor GUS activity. Scale bar: 100 μm.

(B) Longitudinal confocal optical sections of the regions along the primary root shown in (A). Ep: epidermis; Co: cortex; St: stele. The indications upper, middle and lower refer to the a, b and c zones indicated in pane lA. Scale bar: 50 μm.

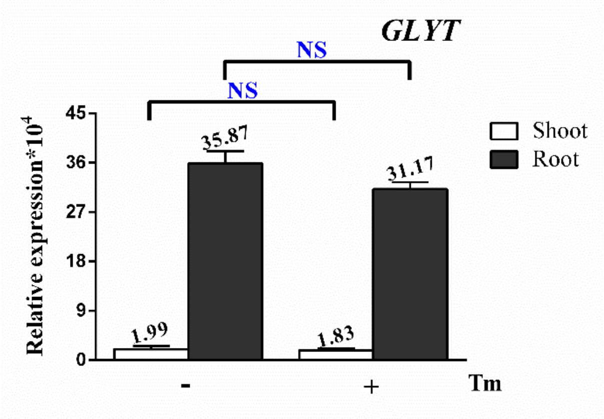

Supplementary Figure 4. *pRoot* drives expression of *GLYT* specifically in the root

qRT-PCR analyses of *GLYT* expression in shoot and root of 14-day-old wild-type (Col-0) seedlings after treatment with DMSO (-) or 0.5µM Tm (+) for 24 hrs. *UBQ10*, *ACT8* and *IPP2* were used as the internal controls.

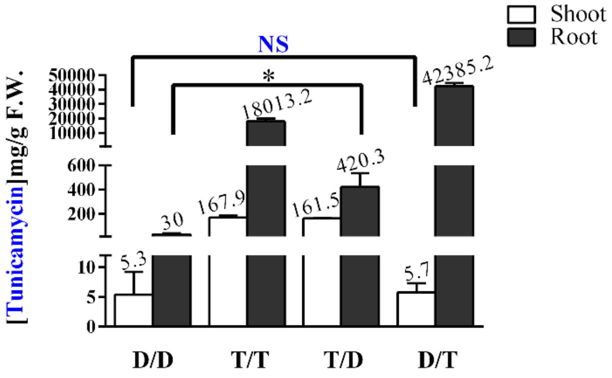

Supplementary Figure 5. Distribution of Tm in *bzip28/60*

Quantitative HPLC/MS analyses of Tm content in shoot and root of *bzip28/60* seedlings after treatment in shoot-root split culture system[2] with 0.5μM Tm or DMSO (Tm control) for 24 hrs. In this system, intact seedlings are laid over Petri dishes that are subdivided by a sealed plate divider. Each dish sub-compartment contains growth medium. The shoot and root portions of intact seedlings are placed across the plate divider and are therefore exposed to the medium contained in each plate sub-compartment separately. D/D denotes both shoot and root on DMSO-containing media; T/T denotes both shoot and root on Tm-containing media; T/D denotes shoot on Tm-containing medium and root on DMSO-containing medium; D/T denotes shoot on DMSO-containing medium and root on Tm-containing medium. The numbers over the histograms express ng g^{-1} fresh weight (F.W.). Data significantly different from the corresponding control are indicated (*$P < 0.05$, NS, non significant; Unpaired t-test).

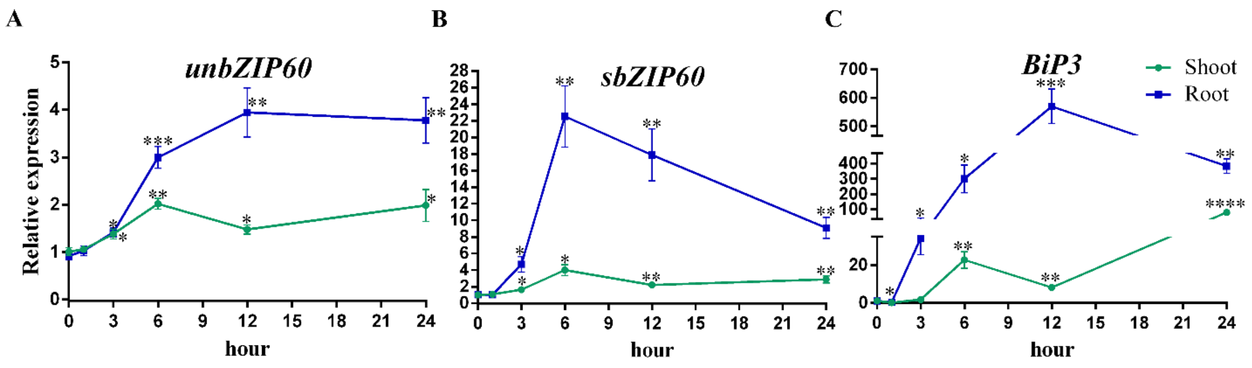

Supplementary Figure 6. Transcriptomic kinetic response of systemic UPR signaling

(A, B, C) Quantitative RT-PCR analyses of *unbZIP60* (A), *sbZIP60* (B) and *BiP3* (C) in 14-day-old wild-type seedlings treated with DMSO or 0.5μM Tm at roots only for the indicated times in the shoot-root split culture system. Values are presented relative to non-treated control (0 hr), which was set to 1. Error bars represent s.e.m among three biological replicates. Data significantly different from the corresponding control (0 hr) are indicated by asterisks (*P < 0.05, **P<0.01, ***P<0.001, ****P<0.0001; Unpaired t-test).

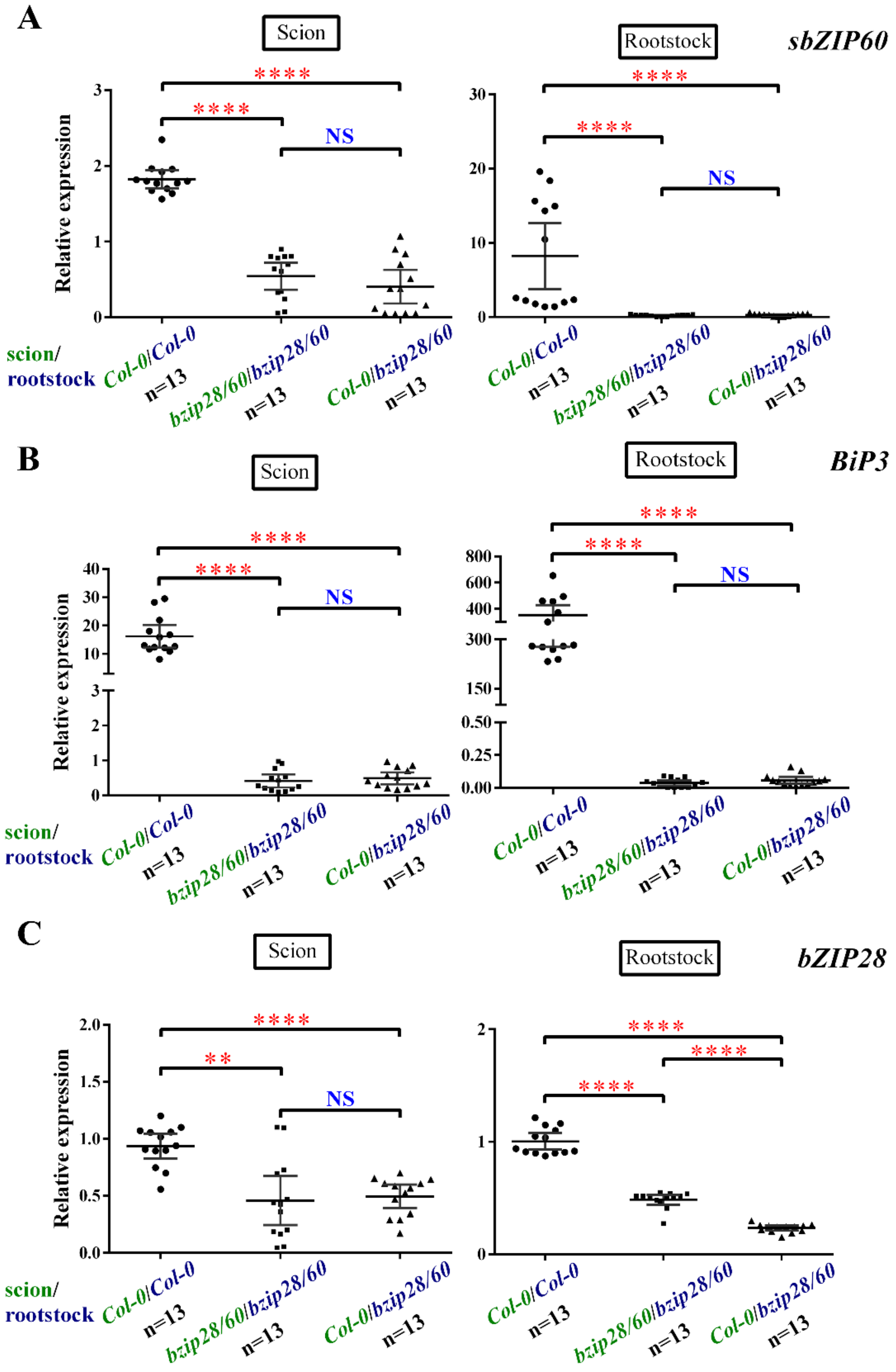

Supplementary Figure 7. Assessment of endogenous systemic transcripts in the plant UPR

(A, B, C) Plots of the mean values (dots) of the quantitative RT-PCR analyses of the transcripts of *sbZIP60* (A) *BiP3* (B) and *bZIP28* (C) in scion and rootstock of 4-week-old grafted seedlings of

wild-type and *bzip28/60* backgrounds treated with DMSO or 0.5μM Tm for 48 hrs as described in Fig. 3A. The average of all the values is indicated by a black line. Error bars represent mean with 95% confidence interval among all grafts. Error bars above and below indicate the 95th and 5th percentiles. Average values significantly different from the values of wild-type (Col-0) and *bzip28/60* self-grafts are indicated by red asterisks (**P<0.01, ****P<0.0001, NS, non significant; Mann-Whitney test). n, total number of grafted unions.

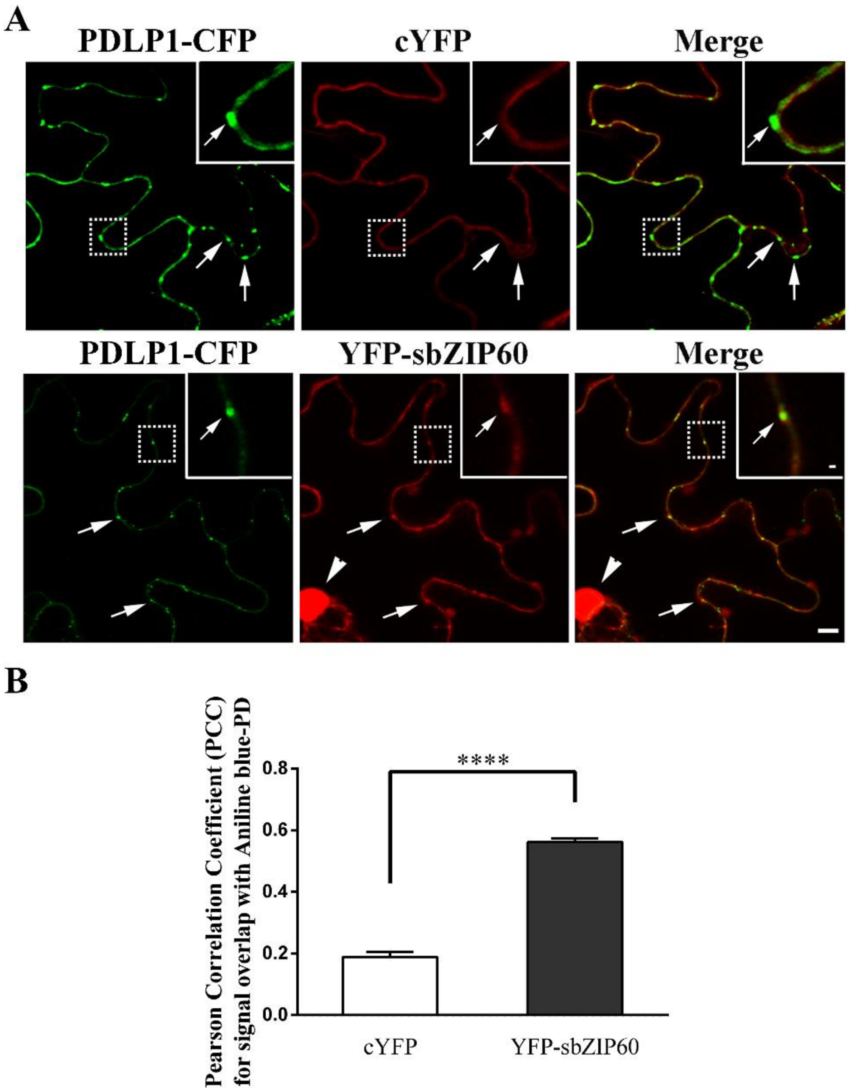

Supplementary Fig. 8. PD-associated bZIP60 is a mobile transcription factor moving intercellularly

(A) Subcellular distribution of cytosolic YFP (cYFP, cytosolic control) and YFP-sbZIP60 in *N. tabacum* leaf epidermal cells co-expressing the PD marker PDLP1-CFP. Similar to PDLP1-CFP, cYFP was distributed uniformly in the cytosol but PDLP1-CFP also accumulated at PD (arrows). The subcellular localization of YFP-sbZIP60 overlays that of PDLP1-CFP but YFP-sbZIP60 also localized to the nucleus (arrowhead). Scale bar: 10 µm.

(B) Quantification analyses of PD visualized by AB with either cYFP or YFP-sbZIP60. The graph represents the extent of overlay between AB at PD and YFP fusion proteins obtained by calculating the Pearson correlation coefficient (PCC). A total of 170 independent PD for each combination were estimated.

Supplementary Table 1. Primers used in this work

Primer name	Sequence	Purpose
UBQ10 For	5'-GGCCTTGTATAATCCCTGATGAATAAG-3'	qRT-PCR
UBQ10 Rev	5'-AAAGAGATAACAGGAACGGAAACATAGT-3'	qRT-PCR
ACT8 For	5'-TCAGCACTTTCCAGCAGATG-3'	qRT-PCR
ACT8 Rev	5'-ATGCCTGGACCTGCTTCAT-3'	qRT-PCR
IPP2 For	5'-GTATGAGTTGCTTCTCCAGCAAAG-3'	qRT-PCR
IPP2 For	5'-GAGGATGGCTGCAACAAGTGT-3'	qRT-PCR
bZIP60us_FWD	5'-GGAGACGATGATGCTGTGGCT-3'	qRT-PCR
bZIP60s_REV	5'-CAGGGAACCCAACAGCAGACT-3'	qRT-PCR
BiP3 For1	5'-CGAAACGTCTGATTGGAAGAA-3'	qRT-PCR
BiP3 Rev1	5'-GGCTTCCCATCTTTGTTCAC-3'	qRT-PCR
bZIP28 For1	5'-CGTCATCAGTCTCCAGCATTTC-3'	qRT-PCR
bZIP28 Rev1	5'-CTTGCCGTGGGTAGTGACATT-3'	qRT-PCR
GLY T For	5'-TCCGACGTTGAGACCACAGG-3'	qRT-PCR
GLY T Rev	5'-GCCACGACAGGTTTCCCACA-3'	qRT-PCR
pSHR F1	5'-ATGTTTTGAAAATTAGTCTGGATCTGAAATTCTTTAATTAGC-3'	Promoter amplification for construct in pSHR-sbZIP60-GFP line
pSHR R1	5'-AAATTCCTCCGCCATTGAATAGAAGAAAGGGA-3'	Promoter amplification for construct in pSHR-sbZIP60-GFP line
sbZIP60 F1	5'-TCCCTTTCTTCTATTCAATGGCGGAGGAATTT-3'	CDS amplification for construct in

		pSHR-sbZIP60-GFP line
bZIP60 R4	5'-ACGCCGCAAGGGTTAAGATTTGGTAT-3'	CDS amplification for construct in pSHR-sbZIP60-GFP line
BiP3 pro F1	5'-TACAATTACATTATTCACGCTG-3'	Promoter amplification for construct in pBiP3-GUS line
BiP3 pro R1	5'-TAGTTTATTTGGAAGAGTATGAAGTTC-3'	Promoter amplification for construct in pBiP3-GUS line

Supplementary References.

1. Parra-Rojas, J., Moreno, A. A., Mitina, I. & Orellana, A. The dynamic of the splicing of bZIP60 and the proteins encoded by the spliced and unspliced mRNAs reveals some unique features during the activation of UPR in Arabidopsis thaliana. *PLoS One* **10,** 1–21 (2015).

2. Kronzucker, H. J., Li, G., Li, B., Song, H. & Shi, W. AUX1 and PIN2 protect lateral root formation in Arabidopsis under Fe stress. *Plant Physiol.* **169,** 2608–2623 (2015).
